# Obsessive–compulsive symptoms as a unique presentation of complex posttraumatic stress disorder in Southeast Asia: a case report

**DOI:** 10.1186/s13256-025-05323-x

**Published:** 2025-05-31

**Authors:** Amanda Albert, Yoke Yong Chen

**Affiliations:** Faculty of Medicine and Health Sciences, Universiti Malaysia Sarawak (UNIMAS), Jalan Datuk Mohd Musa, 94300 Kota Samarahan, Sarawak Malaysia

**Keywords:** Case report, Obsessive–compulsive, Complex posttraumatic stress disorder, OCD, PTSD, CPTSD, Southeast Asia

## Abstract

**Background:**

Posttraumatic stress disorder is a mental health condition outlining psychological sequelae experienced after encountering a traumatic event. Complex posttraumatic stress disorder, however, is increasingly recognized as being distinct from posttraumatic stress disorder. This is due to an observed variation from what is traditionally defined as a traumatic event, as well as greater heterogeneity in its presentation. Cultural factors may also influence definitions of traumatic events and heterogeneity in presentation.

**Case presentation:**

In this case, a 27-year-old Malay male presented with a 9-year history of obsessive–compulsive symptoms of predominantly sexual content. Although initially treated as obsessive–compulsive disorder, persistent negative self-image and features of complex posttraumatic stress disorder surfaced in the course of therapy, stemming from a culturally-related punitive upbringing as well as bullying by peers. He responded markedly well to trauma-based psychotherapy and remains well at time of writing.

**Conclusion:**

A diagnosis of complex posttraumatic stress disorder should be considered in the individual who presents with mental health difficulties, particularly if the individual’s symptoms are atypical to classical diagnostic criteria or the individual does not respond to conventional treatment. It is important to note the role of cultural background—this may give rise to unique presentations of complex posttraumatic stress disorder, and the triggering events may not be traditionally defined as traumatic. Cultural background may also potentially inform treatment and future prevention strategies for complex posttraumatic stress disorder.

## Introduction

Posttraumatic stress disorder (PTSD) is a mental health condition diagnosed on the basis of a history of a traumatic event, where one is exposed to actual or threatened death, serious injury, or sexual violence. The World Health Organization’s (WHO) 11th revision of the International Classification of Diseases (ICD-11) describes the event or series of events as “extremely threatening or horrific” [1]. According to the 5th edition of the Diagnostic and Statistical Manual of Mental Disorders (DSM-V), the diagnostic criteria for this disorder include four compulsory core symptom groups: intrusion; avoidance; negative alterations in cognition and mood; and hyperarousal and reactivity [2]. Direct exposure to the traumatic event is a prerequisite for the diagnosis of PTSD [3].

However, repeated trauma is common, and trauma symptoms can be heterogeneous, as first argued by Herman in 1992 [4, 5]. In 2020, the ICD-11, recognizing the symptom heterogeneity among trauma survivors, introduced a new diagnostic category called complex PTSD (CPTSD). CPTSD is a term used to define the complex presentations that extend beyond the classical symptoms of PTSD. The diagnostic criteria for CPTSD include, in addition to classical PTSD symptoms, additional disturbances in three key domains of “Disturbances of Self-Organisation” (DSO): affective dysregulation, negative self-concepts, and relationship disturbances [1]. This diagnosis has yet to be listed in the DSM-V. The trajectory of PTSD and CPTSD can be highly variable. Predicting who will go on to develop the disorder is a challenge in itself, and causes are likely multifactorial [6].

The lifetime prevalence of CPTSD lies at 1–13% globally, but rates can increase to 50% within mental health service settings. [5] This is reflected similarly in several East Asian studies of populations in China, Hong Kong, Taiwan, and Japan by Ho *et al*. (2019, 2022), which found general population rates of 3.6–10.63% and clinical population rates of up to 55% [7–9]. However, at this point, there still remains a scarcity of research specifically on the presentation or symptom profile of CPTSD in Southeast Asia—the region of Asia consisting of Thailand, Myanmar, Laos, Vietnam, Cambodia, Singapore, the Philippines, Indonesia, Brunei, and Malaysia [10, 11].

Obsessions are unwanted and intrusive recurrent and persistent thoughts, urges, or images; whereas compulsions are repetitive behaviors that an individual is driven to perform in response to obsessions [2]. Apart from being presenting symptoms of obsessive–compulsive disorder (OCD), obsessive–compulsive symptoms are known presenting symptoms of other conditions such as Wilson’s disease [12]. A previous case report has reported association with conditions such as asymmetric temporal lobe atrophy [13]. Research around the comorbidity of OCD and PTSD has found greater rates of sexual obsessive–compulsive symptoms and greater severity of anxiety and depressive symptoms in patients who develop OCD after PTSD. There is also greater functional impairment in individuals with OCD and previous exposure to trauma [14–16]. There is even suggestion of a posttraumatic subtype of OCD [15]. However, most of these studies focus on PTSD as traditionally defined in response to a significant major life event, and not specifically CPTSD.

Looking specifically at CPTSD and its intersection with OCD, D’Angelo *et al*. (2024) examined the coexistence of CPTSD from prolonged interpersonal trauma in individuals with diagnosed OCD. They found greater exacerbation of OCD symptoms in such individuals [17]. In addition, childhood trauma has been repeatedly linked with the development, progression, and severity of both CPTSD and OCD [16, 17]. It is also interesting to note that this may differ according to the subtype of trauma—physical abuse, physical neglect, and emotional abuse are associated with increased obsessive–compulsive symptom severity [17–19]. To date, there have been no network-based studies on the psychopathological overlap between these two disorders, but intrusive thoughts, imagery, or memories are known to be key central features in both disorders [20].

Neurobiologically, it is established that the amygdala-hippocampus-medial prefrontal circuit plays a major role in threat-related behaviors. Multiple studies have found a general reduction in grey matter volume in individuals with PTSD, with some studies indicating a different pattern of reduction on the basis of whether trauma is single-incident or prolonged [21–24]. Studies of the brains of individuals with PTSD or complex PTSD indicate an impairment in hippocampal and dorsolateral prefrontal cortex, as well as an increase in activity in the amygdala and the part of the prefrontal cortex involved in threat expression [6, 25, 26]. Impairment in hippocampal circuits is found in individuals with the FKBP5 polymorphism, a known polymorphism linked to PTSD [26, 27]. Impaired hippocampal circuits may even be a predictor of the longitudinal persistence of PTSD [28].

More recently, further work by Bryant *et al*. (2021) has provided the first evidence of distinct neural profiles differentiating CPTSD from PTSD during threat processing, namely greater bilateral insula and right amygdala activation [25]. This is consistent with disturbances in emotional regulation and self-concept that are prominent in individuals with CPTSD. Current scientific theories suggest that this neurobiological predisposition to CPTSD, combined with other predisposing factors such as genetic vulnerabilities and obsessive–compulsive tendencies, may interact with childhood trauma to give rise to obsessive–compulsive symptoms in adulthood [16, 17, 29]. Other neurobiological studies have also highlighted the roles of neurotransmitters (such as serotonin, norepinephrine, and dopamine) as well as other signaling molecules (such as brain-derived neurotrophic factor (BDNF)) in modulating trauma-related responses in both OCD and PTSD [20, 30–33]. However, these are not sufficient or specific biomarkers for diagnosis, or for distinguishing between the two conditions. There are also no established biomarkers for CPTSD at this point.

To our knowledge, there is no prior research around obsessive–compulsive symptoms being the initial major presenting symptom of CPTSD. Differentiating between primary OCD and CPTSD-related obsessive–compulsive symptoms at this point is therefore primarily by clinical assessment, with the aid of instruments such as the International Trauma Questionnaire (ITQ) that assess CPTSD according to the ICD-11.

Here we present the case of a young man diagnosed with CPTSD who initially presented with obsessive–compulsive symptoms of predominantly sexual content. This report aims to highlight an unusual presentation of CPTSD and underscore the importance of trauma-informed assessment of individuals who present with mental health difficulties.

## Case presentation

### History

Don (not his real name), a 27-year-old Malay male professional, presented to psychiatric services for the first time with a 9-year history of taking photographs and videos of women without their consent. The media was used for masturbation several times a day. He found these behaviors extremely distressing. However, if he did not perform them, he would experience severe anxiety, restlessness, and inability to concentrate at work until it was done. He felt intense sexual gratification during the act, followed by immediate guilt and self-hatred after ejaculation.

These behaviors eventually escalated to the point that he moved from taking photographs or videos in secret to taking them even in the presence of his spouse. Although he initially began with photographs of strangers, he gradually began taking photographs and videos of family members. He would also text other women he knew to obtain their photographs. This culminated in a final event where his spouse found his photographs of a family member and thus threatened divorce. At this point, he decided to seek professional help. He had intense feelings of remorse and was intent on salvaging his relationships with his spouse and her family.

Upon further history, underlying these behaviors were strong obsessional thoughts relating to a fear of sexually assaulting others. He described the behaviors as compulsions—to prevent his obsessions from happening, he had to perform the compulsions. Performing the compulsions caused immediate relief to his anxiety. If the compulsions were not performed, he would experience impairment in his social and occupational functioning.

This presentation was associated with intense feelings of shame, guilt, and self-hatred. He held strong negative views of himself, such as “I am worthless,” “I am a bad person,” and “I do not deserve anything good.” He often struggled with suicidal thoughts. He believed that it was better if he died before he harmed others. The beliefs were strongly held despite attempts to challenge them. However, there were no other associated features of a depressive illness. There were no features suggestive of an anxiety disorder. There were also no features of psychosis or dissociation. He has no prior history of medical illness or substance abuse. He has no known family history of psychiatric illness.

### Mental state examination and physical examination

At the time of presentation, Don was fully orientated, calm, and cooperative. Rapport was easily established. He reported a slightly anxious mood, and his affect was congruent and appropriate. His speech was spontaneous and relevant and normal in tone, volume, and amount. He reported obsessions of sexual content with accompanying compulsive behaviors, but he did not have any disruption in thought flow. He had no perceptual disturbances and no observable abnormal behavior. Cognitive function was intact. He had insight into his illness, and his judgment at the time of initial assessment was intact. Physical examination was unremarkable. Vital signs, including blood pressure, pulse rate, and temperature, were normal on initial assessment.

### Investigations

His free thyroxine levels were slightly elevated on initial assessment, but this eventually normalized without treatment. Other blood investigations and urine for toxicology revealed no abnormalities. He obtained a Yale-Brown Obsessive Compulsive Scale (Y-BOCS) total score of 32 (severe). Plans for brain imaging to work up for organic brain disease on initial assessment were put on hold in view of coronavirus disease-2019 (COVID-19) restrictions and limitation of hospital services at the time. By the time restrictions had lifted, he had begun to demonstrate significant clinical improvement, and brain imaging was deemed of lesser priority.

### Initial treatment

Don was diagnosed with obsessive–compulsive disorder and was started on oral sertraline 50 mg at night, to be tapered up gradually. He was also commenced on psychotherapy.

### Further progress

The obsessions and compulsions responded promptly to treatment, and sertraline was tapered up to a maximum dose of 200 mg at night. Reconciliation with his spouse commenced several months later. The obsessions and compulsions reduced in frequency and intensity, together with a general improvement in life circumstances. However, the strongly negative views of self that he had remained intense, persistent, and out of proportion to his obsessive–compulsive symptoms. As psychotherapy progressed, the negative self-image showed itself to be a strong overarching issue, dominating all areas of his life.

On longitudinal observation, several events triggered emotional distress in Don. This emotional distress was demonstrated in the form of either relapse of his obsessive–compulsive symptoms, self-harm and suicidal thoughts, or new onset of symptoms never experienced before, such as panic attacks. These associated symptoms were limited to the triggering events, and none arose to the level of a separate comorbid psychiatric disorder. During these episodes, there would be an accompanying exacerbation of his self-hatred and self-blaming. These triggering events included his wife’s pregnancy, the initial period after the birth of his child, and receiving news of a change of therapist that turned out to be temporary. These episodes of emotional distress were a surprise to Don himself, as he did not expect to experience those feelings for those events.

Don was initially commenced on exposure and response prevention (ERP) therapy given obsessive–compulsive symptoms. However, considering the above developments, the focus was shifted to mindfulness-based and eye movement desensitization and reprocessing (EMDR) therapy for subsequent sessions, followed by imaginary rescripting and psychodynamic psychotherapy. During these sessions, previously repressed childhood trauma was brought to the conscious stage: frequent belittling criticism from his mother; physical punishment from his father over trivial childhood play; constant feelings of being trampled and suppressed by an authoritarian parenting style; frequent feelings of “not fitting in”; bullying by school peers; and feeling isolated and sidelined as a child, both at home and at school. The prolonged and repetitive manner of these cumulative traumas, together with the difficulties with self-identity and relational capacity, led to a revised diagnosis of CPTSD.

Don found that trauma-based psychotherapy sessions were most helpful, which also lends support to a trauma-related diagnosis. His devotion to his spouse as a close emotional partner, as well as his fatherly instincts after the birth of his child, with a strong desire to protect them both, have served as protective factors in the management of his trauma-related obsessive–compulsive symptoms. The timeline of Don’s follow-up is demonstrated in Table 1.

**Table 1:** Timeline of Don’s follow-up

Time from first visit in week(s)	Significant event
1	Commenced on sertraline and psychotherapy
3	Reported a 50% reduction in obsessive–compulsive symptoms
8	Reconciliation with spouse
13	Sertraline titrated to maximum dose
16	First reported panic attack—related to triggering event
24	Reported reconciliation with spouse’s family
25	Memories of being bullied and sidelined by peers in school surfaced during EMDR, with prominent feelings of “helplessness” and “powerlessness”
48	Reports feeling like his authority is “challenged” by his spouse
66	Recollection of developmental trauma—criticism from mother
98	Had a dream about family’s criticism, memories resurfaced of being physically beaten by father over a trivial matter as a child—linked with strong feelings of “hope crumbling,” despair, uselessness (“better if I did not live since I am a burden to family”)
106	Birth of first child, a son
110	Memories of deeply hurtful criticism by mother surfaced after mother’s comments on his parenting of his newborn son

### Timeline

### Outcome and follow-up

Don remains on pharmacotherapy and ongoing trauma-based psychotherapy and continues to make progress. His roles as a husband and father are empowering, meaningful and protective to him, and his relationships with his extended family have improved. His obsessive–compulsive symptoms are under control. He describes his life as meaningful and a process of continuous self-improvement. He hopes to be able to help others who go through the same challenges.

## Discussion

The presentation of trauma-related issues can be highly heterogeneous. Common presentations include interpersonal difficulties, emotional dysregulation, behavioral issues, low self-esteem, alcohol and drug abuse, anxiety, depression, and even psychosis. [5, 34, 35]. In this case study, we report a more unusual initial presentation of CTPSD, in the form of obsessions and compulsions.

There is growing evidence that PTSD may develop from subjective responses to events not typically defined as traumatic (for example, having invasive procedures performed while going through a major medical event in intensive care), rather than an event that fulfills the conventional definition of “traumatic” [36, 37]. Hyland *et al*. (2020) strongly argue that psychologically threatening events such as bullying and emotional abuse are significantly associated with CPTSD and should be considered traumatic, despite not being defined as a traumatic event in Criterion A of the DSM-V [38]. Bullying has also been linked to greater symptom load in CPTSD in later studies [39, 40]. In other words, it is how an event is subjectively perceived, rather than the objective measure of how traumatic the event typically is, that can trigger and potentiate the trauma response.

In addition, chronic and repeated exposure to traumatic events has been linked with the development of CPTSD [41–43], with significant links specifically to interpersonal trauma [39, 44]. In this report, the patient did not report any single major traumatic event. Instead, he recalled an accumulation of repeated interpersonal traumas over time from relational difficulties with parents and peers (such as criticisms that can be viewed as minor in Asian cultures), starting from early life. This history was not readily available but was only able to be elicited with time, rapport, therapeutic relationship, and the opportune occurrence of certain events. As the trauma was addressed in therapy, a reduction in the frequency and intensity of the obsessions and compulsions as well as an improvement in quality of life followed.

Asian cultural norms of modesty, humility, and emotional restraint may lead to negative self-identity being more commonly endorsed in Asian cultures. Previous research has found that Asians are less likely to self-enhance, more likely to view themselves less positively compared with their Western counterparts, and more likely to view themselves as malleable and changing according to the needs of others [11, 45, 46]. Some of the earliest cross-cultural work among Southeast Asian refugees describes a tendency among Southeast Asian individuals to assume a shy, unassertive, and unemotional display, especially in the presence of healthcare professionals outside of their own culture [47]. Many patients in Southeast Asian cultures, such as the Cambodians written extensively about by Hinton and Bui (1999), may demonstrate a greater degree of somatic symptoms, rather than the predominant psychological symptoms that may be more readily elicited among Western patient populations. There may also be cultural attribution such as “attacks by spirits” or “weakness of one’s own spirit” to specific symptoms experienced in PTSD, and awareness of cultural syndromes is necessary to have meaningful discussions about these experiences [11, 48].

In addition, authoritarian parenting styles are practiced widely in Asian cultures. [49]. Incidence rates of children experiencing violence (including physical and mental violence) have been found to be high in Asia. The practice of “violent discipline” is very common, with rates reported at up to 70% in some countries in Southeast Asia (including Malaysia, the location for this case study). Apart from being viewed as “discipline,” these practices are often also accepted as “tradition.” [50–54] Several Chinese studies have indicated that there are higher levels of child maltreatment among populations in China, as emotional neglect, emotional abuse, and physical abuse are often considered effective parenting strategies [9, 55–57]. Parents and educators are at times described as expressing love using “verbal violence,” [9] that is, harsh criticisms with good intentions. At times, this extends to self-criticism, which is viewed as a way to motivate oneself to improve or to adjust to the environment [11]. This may influence response to negative self-identity (for example, “I feel worthless”), and the Asian individual may find it confronting to address emotional needs directly.

These unfulfilled emotional needs and deep emotional pain may instead be directed inwardly [9, 57, 58] or may be expressed via dissociation, as significantly demonstrated by Wang *et al*. (2024) in a large study of trauma-exposed high school students in China [59]. They may also be expressed in other more covert but extreme outlets such as suicidality and voyeuristic-like or obsessive–compulsive behaviors. The initial presentation for this patient appeared to be sexually obsessive–compulsive, and the link to CPTSD and a deep emotional need only emerged after a gradual process of building a therapeutic relationship and addressing his prominent and disproportionate negative self-image. This is consistent with European cross-cultural network studies that found “feelings of worthlessness” to be the most central symptom in the network for CPTSD [60], in line with the predominant issue that surfaced during this patient’s psychotherapy sessions. In addition, in some early work on PTSD in Southeast Asian refugee populations, almost half were diagnosed with PTSD only after several years in treatment. Thus, to accurately determine the presence of PTSD in Asian populations, a sensitive interview in a therapeutic situation over time is recommended [47]. Treatment may require a longer course, a greater number of psychological interventions, or a more gradual stepped pace of intervention. To date, there is no comprehensive work carried out on CPTSD in Southeast Asia that is sufficient to make clinical recommendations specifically for Southeast Asian populations.

The association between CPTSD and negative self-identity with childhood adversities are consistent with previous findings from Western cultures [61]. Our case study indicates that CPTSD may also present with intense feelings of guilt and shame, and a strong disapproval of self by self and the community, particularly in the Asian collectivist culture. This contrasts with Western culture, which takes a more individualistic approach to life and problem-solving. The impact of collective community disapproval in the Asian community is further compounded by findings that lack of perceived social support has been significantly linked with the development and maintenance of CPTSD [39, 62]. This may introduce unique challenges in the treatment of CPTSD in Asian communities. More research into different culturally-informed clinical approaches is required.

CPTSD is a newly adopted diagnosis, and there are no clinical studies to date addressing benefits of specific treatments. Current studies recommend building upon previous well-established PTSD treatments such as cognitive behavioral therapy (CBT) and eye movement desensitization therapy (EMDR) with a combination of treatment components that include self-regulation strategies and trauma-focused interventions [5, 63, 64]. Strengthening capacity for self-regulation and addressing underlying trauma is a major part of the therapeutic intervention for CPTSD in both adults and adolescents [17, 65]. Psychosocial factors can play a role in maintaining CTPSD; therefore, structured psychoeducation and intervention sessions involving close cohabiting individuals in the patient’s life are also recommended to address factors that may perpetuate ongoing trauma [17]. In Asian cultures, this may be extended to address cultural aspects such as “verbal violence” in the parent–child relationship, which perpetuates trauma even when the child is an adult.

Furthermore, strengthening the parent–child relationship may have implications for the prevention of childhood trauma as well. There is a growing body of evidence for the effectiveness of culturally-adapted parenting interventions in reducing violence (including “violent discipline”) toward children in Southeast Asia. These interventions have largely been based on social learning theory. Such interventions not only reduce violence toward children but also reduce behavioral problems in children and increase parental efficacy and positive parenting [50, 66]. Some studies have even noted a secondary outcome of reducing intimate-partner violence between parents, which would also have positive effects on child well-being and possibly prevent the development of CPTSD [50].

From the healthcare provider perspective, an interesting finding from a study of adults who had engaged with traditional Chinese medicine practitioners reports that individuals who perceived their practitioners to be more modern (endorsing or practicing concepts such as egalitarianism) reported lesser CPTSD symptoms [67]. While preliminary, this is certainly worth expanding on to consider the role of different kinds of healthcare practitioners in various cultures in the treatment of CPTSD in Southeast Asia. Overall, these considerations may form a vital culture-specific part of the treatment regime suggested by the International Society for Traumatic Stress Studies (ISTSS)—flexible selection and implementation of multiple components of treatment to create a personalized, patient-centered regime relevant to each individual patient [64].

The most robust evidence for the role of serotonin in both OCD and PTSD arises from their common denominator in the use of selective serotonin reuptake inhibitor (SSRI) antidepressants for treatment. Even then, the role of pharmacotherapy for PTSD is limited, with some research existing for a few of the SSRI antidepressants (fluoxetine, paroxetine, and sertraline) and one serotonin–norepinephrine reuptake inhibitor (venlafaxine). Meta-analyses indicate that effect sizes are small, and, thus, these are not universally approved according to clinical guidelines. They are not recommended as first-line treatment (6, 68). It therefore remains to be seen whether pharmacotherapy will play a role in the management of CPTSD. However, it may be prudent to build on current recommendations for clinical practitioners to utilize pharmacotherapy to co-manage posttraumatic, anxiety, or depressive symptoms arising from CPTSD that cannot be managed by psychotherapy alone or in other specific clinical situations. This is the management approach in this case study, with good effect.

## Conclusion

This case study demonstrates a unique presentation of CPTSD, in the form of obsessive–compulsive symptoms. It sheds some useful insights on the heterogeneous presentation of this newly included diagnosis in the ICD-11, particularly in the context of Asian cultural norms. Using a personalized patient-based approach and utilizing existing practice for the treatment of PTSD led to marked progress in the reduction of the patient’s symptoms as well as improvement in interpersonal relationships and quality of life. We hope to highlight the need for early suspicion of the diagnosis of CPTSD when encountering patients with a history of childhood trauma and features such as strong negative self-identity, regardless of initial presentation. We also hope to spark further interest in the role of Southeast Asian cultural norms in the development and maintenance of CTPSD, consequently informing culturally-sensitive treatment and prevention of the disorder.

## Data Availability

Data sharing is not applicable to this article as no datasets were generated or analyzed during the current study.

## Abbreviations

CPTSD: Complex posttraumatic stress disorder
DSM-V: Diagnostic and Statistical Manual of Mental Disorders, Fifth Edition
ICD-11: International Classification of Diseases, 11th Revision
OCD: Obsessive–compulsive disorder
PTSD: Posttraumatic stress disorder
